# Country of birth and non-small cell lung cancer incidence, treatment, and outcomes in New South Wales, Australia: a population-based linkage study

**DOI:** 10.1186/s12890-022-02163-z

**Published:** 2022-09-27

**Authors:** Alana Little, David Roder, George W. Zhao, Sheetal Challam, Ashanya Malalasekera, David Currow

**Affiliations:** 1 Cancer Information and Analysis, Cancer Institute New South Wales, St Leonards, NSW 2065 Australia; 2 Equity, Multicultural Program, Cancer Institute New South Wales, St Leonards, NSW 2065 Australia; 3 Faculty of Medicine, The University of Sydney, Sydney, NSW 2006 Australia; 4 Health and Sustainable Futures, University of Wollongong, Wollongong, NSW 2500 Australia

**Keywords:** Lung cancer, Country of birth, Australia, Incidence, Treatment, Survival

## Abstract

**Objective:**

To compare treatment within 12 months of diagnosis, and survival by country of birth for people diagnosed with invasive non-small cell lung cancer (NSCLC) in New South Wales (NSW), Australia.

**Design, patients, and setting:**

A population-based cohort study of NSW residents diagnosed with NSCLC in 2003–2016 using de-identified linked data from the NSW Cancer Registry, NSW Admitted Patient Data collection, Emergency Departments, Medicare Benefits and Pharmaceutical Benefits Scheme, and National Death Index.

**Main outcome measures:**

Odds of receiving any treatment, surgery, systemic therapy, or radiotherapy respectively, in the 12 months following diagnosis were calculated using multivariable logistic regression. The hazard of death (all-cause) at one- and five-years following diagnosis was calculated using multivariable proportional hazards regression.

**Results:**

27,114 People were recorded with NSCLC in the 14-year study period. Higher percentages of older males from European countries applied in the earlier years, with a shift to younger people from South East Asia, New Zealand, and the Middle East. Adjusted analyses indicated that, compared with the Australian born, people from European countries were more likely to receive treatment, and, specifically surgery. Also, people from Asian countries were more likely to receive systemic therapy but less likely to receive radiotherapy. Survival at one- and five-years following diagnosis was higher for people born in countries other than Australia, New Zealand the United Kingdom and Germany.

**Conclusions:**

Variations exist in treatment and survival by country of birth in NSW. This may be affected by differences in factors not recorded in the NSW Registry, including use of general health services, family histories, underlying health conditions, other intrinsic factors, and cultural, social, and behavioural influences.

**Supplementary Information:**

The online version contains supplementary material available at 10.1186/s12890-022-02163-z.

## Introduction

**Fig. 1:**
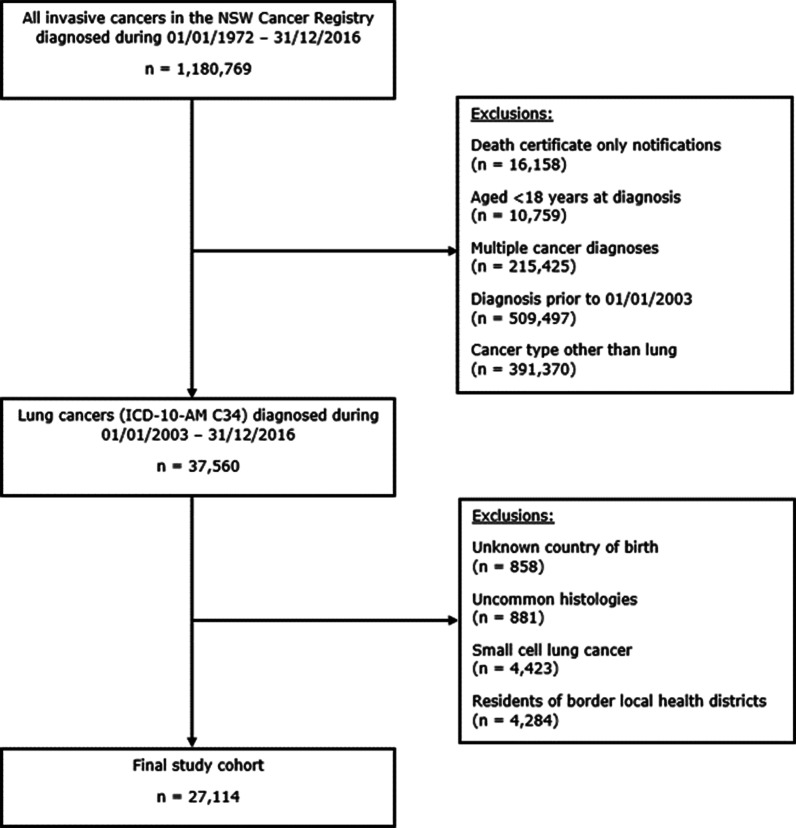
Cohort selection diagram

**Fig. 2:**
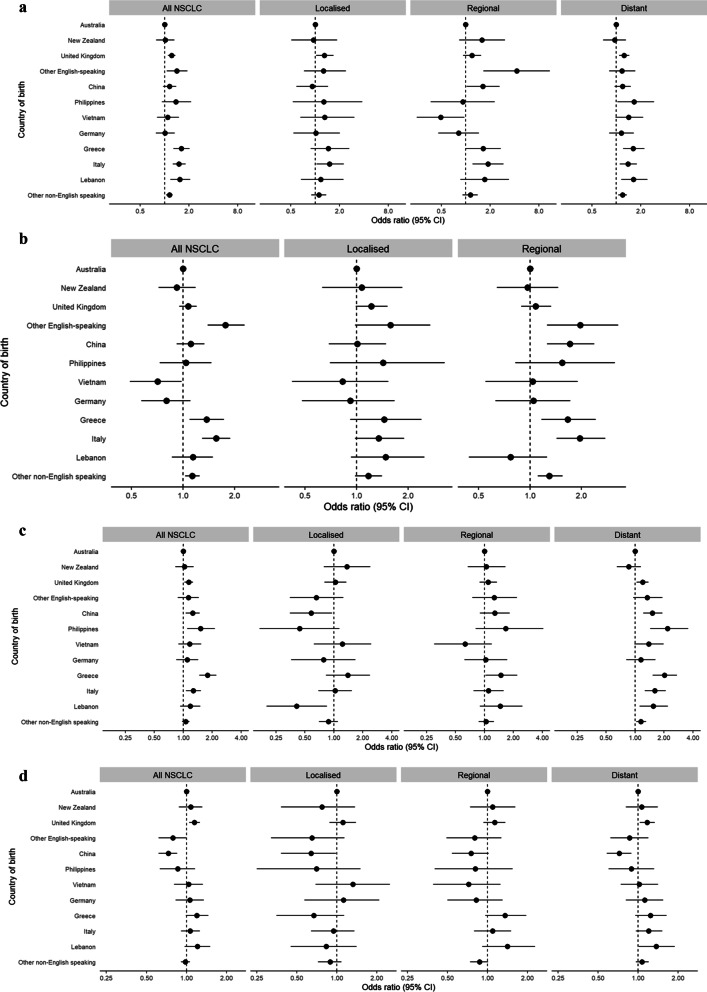
**a** Forest plot for receipt of any treatment within 12 months of diagnosis, NSCLC, 2003–2016. **b** Forest plot for receipt of surgery within 12 months of diagnosis, NSCLC, 2003–2016. **c** Forest plot for receipt of systemic therapy within 12 months of diagnosis, NSCLC, 2003–2016. **d** Forest plot for receipt of radiotherapy within 12 months of diagnosis, NSCLC, 2003–2016. *Notes*: 1. Dots represent the adjusted odds ratio for each characteristic, with 95% confidence intervals shown as horizontal bars. 2. Each characteristic has a reference value at OR = 1.00

Lung cancer is the leading cause of cancer death for males and females in NSW, with similar age-standardised incidence, mortality and survival outcomes to corresponding national figures [1, 2]. For lung cancers diagnosed in Australia in 2013–2017, the five-year relative survival was 20%. While this survival is low compared to most other cancer types, it represents an absolute increase of 12% in corresponding five-year relative survival since 1988–1992 [2].

Australia’s diverse, multicultural population is reflected in NSW where the percentage of people born outside of Australia increased to 28% in 2016, with 25% of that population mainly speaking a language other than English at home [3]. During 2006–2016, the largest increases in population size applied to migrants from China, India, Nepal, the Philippines, Vietnam, South Korea, and Lebanon [4].

Previous studies in NSW and other Australian jurisdictions have indicated migrants to generally have lower overall cancer incidence and mortality rates than the Australian-born, although differences applied by cancer type and country of birth [5–7].

We aim in this study to determine whether differences exist in NSW between people diagnosed with invasive non-small cell lung cancer (NSCLC) during 2003–2016, in treatment and survival by country of birth. We used analyses adjusted for the baseline characteristics including age at diagnosis, sex, year of diagnosis, comorbidity prevalence, and socioeconomic status.

## Methods

### Data sources

This is a population-based cohort study using linked cancer registry, treatment and death data. The analysis used linked diagnostic data for invasive NSCLC from the NSW Cancer Registry (NSWCR) [8], hospitalisation data from the Admitted Patient Data Collection (APDC) and Emergency Department Data Collection (EDDC), claims to the Medicare Benefits Schedule (MBS) and Pharmaceutical Benefits Scheme (PBS), and death-record data from the National Death Index (NDI) [8]. The health care system in New South Wales operates under a public model, with universal taxpayer-funded access to cancer diagnostic and treatment services available to all Australian citizen and permanent residents. Insurance funded health care is also available on an opt-in basis, which allows for the use of private hospitals and private clinicians. Records of taxpayer-funded and insurance payment episodes were included in this study.

Probabilistic, privacy-preserving, person-level linkages of NSW datasets were performed by the Centre for Health Record Linkage (CHeReL), and of Commonwealth datasets, by the Australian Institute of Health and Welfare (AIHW) [9].

Ethics approval was provided by the NSW Population and Health Services Research Ethics Committee (HREC/15/CIPHS/15) and the AIHW Ethics Committee (EO2016/1/224).

### Study population

The study population comprised all NSW residents aged 18+ years diagnosed with primary invasive NSCLC of the lung, bronchus, or trachea (ICD-O-3: C33, C34, excluding morphologies of 8014, 8042, 8043, 8044, and 8045) with histopathological confirmation, during 2003–2016 [10].

Multiple primary cancers, NSCLC recorded from death certificate only, and cancer cases of unknown country of birth were excluded from the cohort. Residents of local health districts adjacent to NSW borders were also excluded as they may have travelled to other jurisdictions for treatments that were not recorded in NSW (Fig. 1) [9].

### Outcome measures

Data on types of treatment received (surgery, radiotherapy, and systemic therapy respectively) during the 12 months from diagnosis (including neoadjuvant therapies), included fact of surgery identified using APDC data; fact of radiotherapy using a combination of APDC and MBS data; and fact of systemic therapy from a combination of APDC, MBS and PBS data (Additional file 1: Table S1) [8].

All-cause survival at one- and five-years post diagnosis was measured from date of diagnosis to date of death or date of last known contact with the health system [11–13]. People without any records in the NSWCR, APDC, EDDC, MBS, PBS, or NDI for a six-month period or longer were treated as lost to follow-up.

### Covariates

Covariates included in multivariable models included sex, age at diagnosis, calendar year of diagnosis, Charlson comorbidity score, and socioeconomic status [8, 14].

Country of birth (COB) was categorised as Australia, China (excluding Hong Kong, Macau, and Taiwan), Germany, Greece, Italy, Lebanon, New Zealand, the Philippines, United Kingdom, and Vietnam, based on the country of birth recorded on the NSWCR (note: apart from Australia, the four largest COB categories were the United Kingdom, China, Italy, and Greece, in decreasing order). These countries represent the ten countries of birth with the highest lung cancer incidence during the study period. All other countries were grouped into “other English-speaking” or “other non-English speaking” categories [8].

Age was analysed as either a continuous or categorical (18–49, 50–59, 60–69, 70+ years) variable. Socioeconomic status of residential address at time of diagnosis was classified using the Index of Relative Socioeconomic Advantage and Disadvantage developed by the Australian Bureau of Statistics [15]. The year of diagnosis was examined as either a continuous variable or by two-year periods ranging from 2003–2004 to 2015–2016. Extent of disease at diagnosis was categorised as localised (confined to the organ or tissue of origin), regional (spread to adjacent organs or tissues, or spread to regional lymph nodes), distant (spread beyond the organ or tissue of origin to distant body sites), or unknown, as used in the NSW Cancer Registry [8]. The Charlson comorbidity score was calculated from APDC codes for morbidity recorded in admissions in the 12 months leading to NSCLC diagnosis [14].

### Statistical methods

Cross tabulations and the Pearson chi-square test; comparisons  were used to describe cohort characteristics for each covariate, with people born in Australia as the reference group [11, 13]. Multivariable logistic regression was used to convert unadjusted results of analyses by COB to percentages adjusted for covariates [11, 13]. These adjusted percentages are used in the text in preference to the raw data.

Multivariable logistic regression was also used to determine odds ratios for treatment (any type, surgery, radiotherapy, and systemic therapy respectively) [11, 13]. All logistic models included country of birth as the principal study factor, with sex, age at diagnosis, year of diagnosis, Charlson comorbidity score, and socioeconomic status as other predictor variables [8]. Separate treatment models were constructed for all NSCLC combined and by extent of disease (localised, regional, and distant) [8]. People with unknown extent of disease at diagnosis were excluded from these analyses.

Multivariable proportional hazards regression models were created to obtain hazard ratios for death (all causes) in the first- and five-year period following diagnosis [8, 11, 13–15]. All proportional hazards models included country of birth as the principal study factor, plus sex, age at diagnosis, year of diagnosis, Charlson comorbidity score, and socioeconomic status as predictor variables [13, 14]. Separate models were constructed for all NSCLC combined and for each extent of disease category (localised, regional, and distant). People with unknown extent of disease at diagnosis were excluded from these analyses. Proportionality assumptions were checked.

Data preparation and analyses were undertaken using SAS 9.4 (SAS Institute Inc, Cary, USA) [11], and R 3.6.3 (R Core Team, Vienna, Austria) [12].

## Results

### Descriptive characteristics

There were 37,560 invasive lung cancers diagnosed in NSW residents during 2003–2016. After applying the study inclusion criteria, 27,114 people with NSCLC were included in the cohort (Fig. 1). Completeness of records for country of birth was high (only 2.2% of records were excluded due to unknown country of birth).

There was a strong male predominance for people born in Greece (87%), Italy (79%), and Lebanon (79%). Compared with the Australian-born, people born in China, Greece, Italy, and the United Kingdom tended to be older (median age > 70 years) while people born in the Philippines, New Zealand, and Vietnam tended to be younger (median age < 70 years).

Number of cases increased as level of disadvantage increased, except for the *other English-speaking countries* category. The prevalence of comorbidities in the year leading to cancer diagnosis varied across countries, ranging from 14% for China to 26% for Italy. More cases were diagnosed in the later time periods, particularly for China (9% of cases diagnosed in 2003–2004 increasing to 20% in 2015–2016), the Philippines (6% of cases diagnosed in 2003–2004 increasing to 22% in 2015–2016), and Vietnam (9% of cases diagnosed in 2003–2004 increasing to 21% in 2015–2016). Table 1 provides a full of summary of the unadjusted demographic characteristics of the study cohort.

**Table 1:** Cohort characteristics

Sex	Australia	China	Germany	Greece	Italy	Lebanon	New Zealand	Philippines	UK	Vietnam	Other English-speaking	Other Non-English
	Reference			#	#	#		#	#			#
Female	7267 (43.8%)	407 (47.3%)	127 (36.7%)	68 (13.2%)	161 (20.9%)	77 (20.9%)	225 (50.1%)	113 (55.9%)	1028 (40.1%)	110 (36.3%)	177 (49.0%)	1267 (33.5%)
Male	9328 (56.2%)	454 (52.7%)	219 (63.3%)	447 (86.8%)	611 (79.1%)	292 (79.1%)	224 (49.9%)	89 (44.1%)	1533 (59.9%)	193 (63.7%)	184 (51.0%)	2513 (66.5%)

Age at diagnosis	Reference	#		#	#		#	#	#	#		
Median age (years)	70	72	72	73	75	67	65	63	73	66	70	70
18–59 years	3184 (19.2%)	201 (23.3%)	43 (12.4%)	40 (7.8%)	43 (5.6%)	84 (22.8%)	150 (33.4%)	77 (38.1%)	284 (11.1%)	95 (31.4%)	57 (15.8%)	704 (18.6%)
60–69 years	4738 (28.6%)	152 (17.7%)	106 (30.6%)	147 (28.5%)	146 (18.9%)	118 (32.0%)	149 (33.2%)	57 (28.2%)	701 (27.4%)	85 (28.1%)	112 (31.0%)	1075 (28.4%)
70–79 years	5281 (31.8%)	280 (32.5%)	116 (33.5%)	211 (41.0%)	338 (43.8%)	116 (31.4%)	109 (24.3%)	37 (18.3%)	875 (34.2%)	69 (22.8%)	129 (35.7%)	1195 (31.6%)
80+ years	3392 (20.4%)	228 (26.5%)	81 (23.4%)	117 (22.7%)	245 (31.7%)	51 (13.8%)	41 (9.1%)	31 (15.3%)	701 (27.4%)	54 (17.8%)	63 (17.5%)	806 (21.3%)

SEIFA^3^	Reference	#		#	#	#			#	#	#	#
Quintile 1	5045 (30.4%)	173 (20.1%)	91 (26.3%)	117 (22.7%)	153 (19.8%)	172 (46.6%)	121 (26.9%)	52 (25.7%)	715 (27.9%)	187 (61.7%)	63 (17.5%)	1047 (27.7%)
Quintile 2	3965 (23.9%)	198 (23.0%)	83 (24.0%)	137 (26.6%)	162 (21.0%)	92 (24.9%)	100 (22.3%)	40 (19.8%)	569 (22.2%)	65 (21.5%)	55 (15.2%)	810 (21.4%)
Quintile 3	3137 (18.9%)	184 (21.4%)	69 (19.9%)	130 (25.2%)	171 (22.2%)	58 (15.7%)	74 (16.5%)	51 (25.2%)	475 (18.5%)	24 (7.9%)	70 (19.4%)	740 (19.6%)
Quintile 4	2412 (14.5%)	141 (16.4%)	58 (16.8%)	93 (18.1%)	195 (25.3%)	29 (7.9%)	79 (17.6%)	34 (16.8%)	415 (16.2%)	14 (4.6%)	80 (22.2%)	581 (15.4%)
Quintile 5	2036 (12.3%)	165 (19.2%)	45 (13.0%)	38 (7.4%)	91 (11.8%)	18 (4.9%)	75 (16.7%)	25 (12.4%)	387 (15.1%)	13 (4.3%)	93 (25.8%)	602 (15.9%)

Charlson score	Reference	#						#		#		#
0	12,472 (75.2%)	742 (86.2%)	270 (78.0%)	399 (77.5%)	575 (74.5%)	285 (77.2%)	362 (80.6%)	171 (84.7%)	1940 (75.8%)	256 (84.5%)	279 (77.3%)	2994 (79.2%)
≥ 1	4123 (24.8%)	119 (13.8%)	76 (22.0%)	116 (22.5%)	197 (25.5%)	84 (22.8%)	87 (19.4%)	31 (15.3%)	621 (24.2%)	47 (15.5%)	82 (22.7%)	786 (20.8%)

Year of diagnosis	Reference	#				#	#					
2003–2004	2192 (13.2%)	79 (9.2%)	42 (12.1%)	64 (12.4%)	103 (13.3%)	39 (10.6%)	38 (8.5%)	12 (5.9%)	334 (13.0%)	26 (8.6%)	53 (14.7%)	427 (11.3%)
2005–2006	2188 (13.2%)	93 (10.8%)	47 (13.6%)	62 (12.0%)	122 (15.8%)	35 (9.5%)	48 (10.7%)	27 (13.4%)	371 (14.5%)	39 (12.9%)	46 (12.7%)	484 (12.8%)
2007–2008	2347 (14.1%)	109 (12.7%)	52 (15.0%)	70 (13.6%)	103 (13.3%)	45 (12.2%)	83 (18.5%)	31 (15.3%)	387 (15.1%)	32 (10.6%)	31 (8.6%)	532 (14.1%)
2009–2010	2444 (14.7%)	110 (12.8%)	54 (15.6%)	86 (16.7%)	115 (14.9%)	52 (14.1%)	58 (12.9%)	28 (13.9%)	408 (15.9%)	48 (15.8%)	68 (18.8%)	560 (14.8%)
2011–2012	2431 (14.6%)	136 (15.8%)	53 (15.3%)	73 (14.2%)	109 (14.1%)	57 (15.4%)	71 (15.8%)	29 (14.4%)	381 (14.9%)	41 (13.5%)	40 (11.1%)	581 (15.4%)
2013–2014	2474 (14.9%)	162 (18.8%)	45 (13.0%)	78 (15.1%)	111 (14.4%)	82 (22.2%)	82 (18.3%)	31 (15.3%)	333 (13.0%)	55 (18.2%)	57 (15.8%)	569 (15.1%)
2015–2016	2519 (15.2%)	172 (20.0%)	53 (15.3%)	82 (15.9%)	109 (14.1%)	59 (16.0%)	69 (15.4%)	44 (21.8%)	347 (13.5%)	62 (20.5%)	66 (18.3%)	627 (16.6%)

Extent of disease	Reference						#			#		#
Localised	3073 (18.5%)	138 (16.0%)	51 (14.7%)	81 (15.7%)	173 (22.4%)	76 (20.6%)	65 (14.5%)	36 (17.8%)	449 (17.5%)	47 (15.5%)	76 (21.1%)	684 (18.1%)
Regional	3433 (20.7%)	177 (20.6%)	74 (21.4%)	134 (26.0%)	165 (21.4%)	79 (21.4%)	109 (24.3%)	38 (18.8%)	511 (20.0%)	50 (16.5%)	74 (20.5%)	790 (20.9%)
Distant	7630 (46.0%)	428 (49.7%)	167 (48.3%)	234 (45.4%)	336 (43.5%)	179 (48.5%)	235 (52.3%)	112 (55.4%)	1225 (47.8%)	174 (57.4%)	160 (44.3%)	1831 (48.4%)
Unknown	2459 (14.8%)	118 (13.7%)	54 (15.6%)	66 (12.8%)	98 (12.7%)	35 (9.5%)	40 (8.9%)	16 (7.9%)	376 (14.7%)	32 (10.6%)	51 (14.1%)	475 (12.6%)

1. Potential non-random differences are flagged with # (Chi-square test with Bonferroni correction for multiple pair-wise comparisons)

2. SEIFA Quintile 1 = most disadvantaged

The percentage of people lost to follow up at one year following diagnosis varied across countries, from 0.1% for Australia to 5.3% for New Zealand. A similar pattern was seen for loss to follow up at five years following diagnosis, varying from 0.2% for Australia to 6.9% for New Zealand.

### Treatment receipt

After adjusting for age at diagnosis, sex, year of diagnosis, comorbidity score, and socioeconomic status, the percentage of people recorded as having any treatment in the 12 months from diagnosis varied across country of birth categories from 68% for Australia to 76% for Greece (all NSCLC) and from 69% for China to 77% for Italy (localised disease).

Corresponding percentages receiving any treatment within 12 months from diagnosis for more advanced disease varied from: 68% for Vietnam to 93% for other English-speaking countries (regional disease), and from 61% for New Zealand to 71% for the Philippines (distant disease). Compared with the Australian-born, and apart from those born in Germany, those born in most European or other English-speaking countries had higher odds of receiving any treatment. Odds of receiving any treatment was also elevated for people born in Lebanon. While this pattern generally applied for each extent of disease, “statistical significance” was inconsistent. In addition, for distant disease, receipt of any treatment had higher odds for those born in the Philippines and other non-English speaking countries than for the Australian-born. (Fig. 2a, Additional file 1: Table S2).

The adjusted percentage of people who received surgery also varied across countries, ranging from 14% for Vietnam to 29% for other English-speaking countries (all NSCLC), 40% for Vietnam to 53% for other English-speaking countries (localised disease), and 27% for Lebanon to 48% for other English-speaking countries (regional disease). Compared with the Australian-born, those born in Greece, Italy, other English-speaking, and other non-English speaking countries had higher odds of surgery, whereas lower odds applied for those born in Vietnam. While this generally applied for each extent of disease, “statistical significance” was less consistent. In addition, people born in Vietnam had lower odds of surgery for localised disease. (Fig. 2b, Additional file 1: Table S3).

The adjusted percentage of people who received systemic therapy varied across countries, ranging from 37% for Australia to 49% for Greece (all NSCLC); 9% for Lebanon to 24% for Greece (localised disease); 39% for Vietnam to 58% for the Philippines (regional disease); and 37% for New Zealand to 55% for the Philippines (distant disease). Compared with those born in Australia, people from the United Kingdom, China, the Philippines, Greece, and Italy were more likely to receive systemic therapy for all NSCLC, people from Greece were more likely to receive systemic therapy for regional disease, and people from the United Kingdom, China, the Philippines, Greece, Italy, Lebanon, and other non-English speaking countries were more likely to receive systemic therapy for distant disease (Fig. 2c, Additional file 1: Table S4).

The adjusted percentage of people who received radiotherapy varied across countries, ranging from 34% for China to 45% for Lebanon (all NSCLC); 16% for China to 29% for Vietnam (localised disease); 38% for Vietnam to 54% for Lebanon (regional disease); and 38% for China to 53% for Lebanon (distant disease). Compared with those from Australia, people from Asian countries were less likely to receive radiotherapy (except for Vietnam, where the pattern was less clear; and people from European countries who were more likely to receive radiotherapy for all NSCLC, regional disease (except Germany), and distant disease (Fig. 2d, Additional file 1: Table S5).

### Survival

**Fig. 3:**
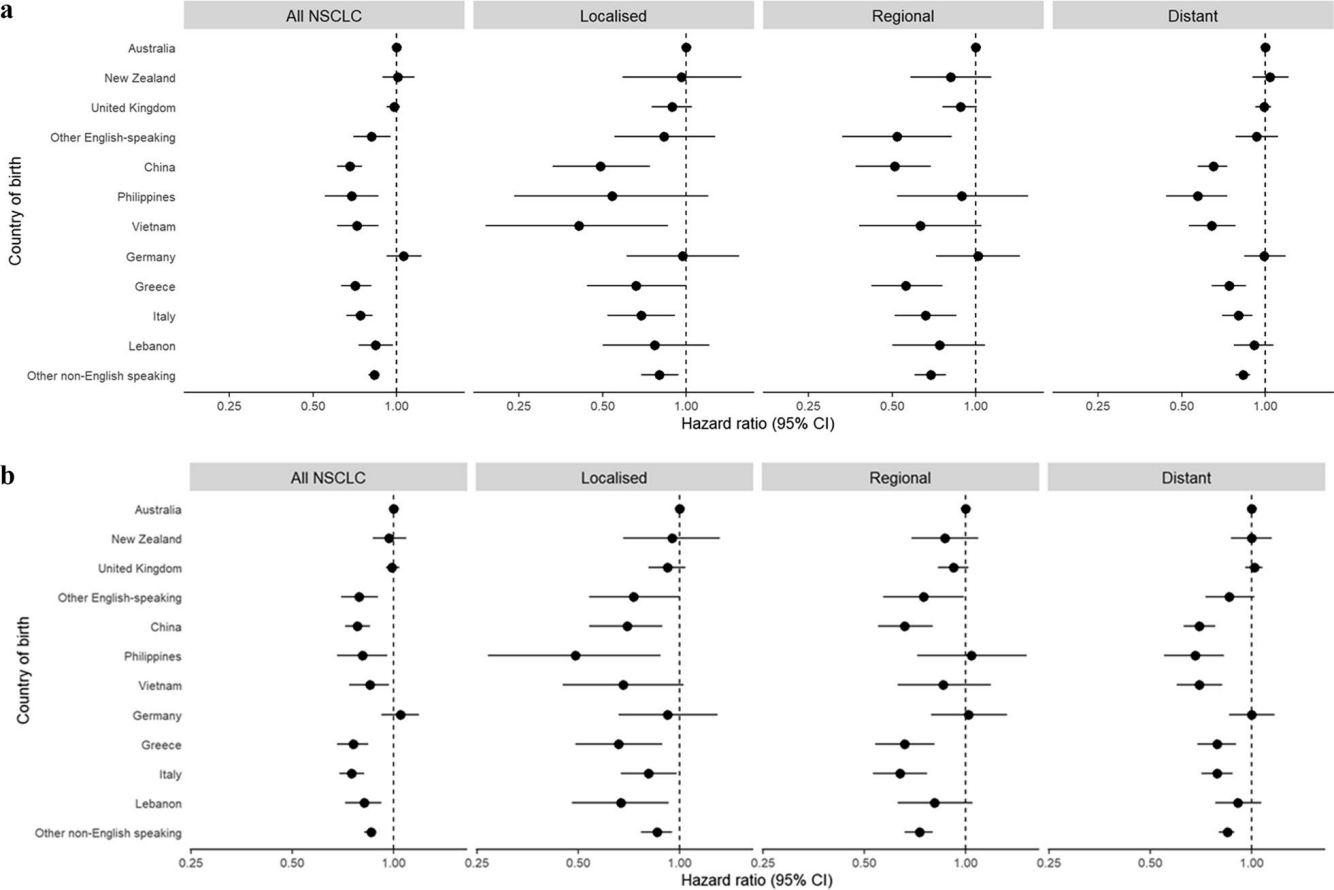
**a** Forest plot for hazard of death at one year following diagnosis, NSCLC, 2003–2016. **b** Forest plot for hazard of death at five years following diagnosis, NSCLC, 2003–2016. *Notes*: 1. Dots represent the adjusted hazard ratio for each characteristic, with 95% confidence intervals shown as horizontal bars. 2. Each characteristic has a reference value at HR = 1.00

Compared to people born in Australia, those from Asian, European (except Germany), other English-speaking countries, and other non-English speaking countries had a reduced hazard of death at one year following diagnosis. This pattern was seen across all extents of disease (Fig. 3a, Additional file 1: Table S6).

Similar results were seen for five-year survival. Compared with people born in Australia, people from Asian, European (except Germany), other English-speaking countries, and other non-English speaking countries had a lower hazard ratio for death in the five years following diagnosis. This pattern was seen across all extents of disease (Fig. 3b, Additional file 1: Table S7).

## Discussion

The sociodemographic characteristics of incidence and outcomes observed in this NSW study of NSCLC align with changing migration patterns, including the higher percentages of older and male cases from European countries in the earlier periods and higher percentages of younger cases from South East Asia, New Zealand, and the Middle East in more recent years [3, 4]. This pattern reflects trends in migration composition from predominately European countries following World War II, and to South East Asia and the Middle East in more recent decades [4].

Services need to account for cultural changes introduced by immigration when inviting people for screening and treatment. People who migrated from Greece, Italy, and Lebanon may have higher rates of tobacco use, especially among males, and may warrant added emphasis in tobacco control programs [16]. It has long been recognized that cultural factors can affect health-service utilization, including readiness to seek medical care in response to symptoms [17]. Health education programs should also address the risks of low screening participation and reluctance to seek treatment that may occur across cultural groups.

We found a higher level of socioeconomic disadvantage in people born in Vietnam and Lebanon, which may affect health-service participation. Socioeconomic disadvantage may compound differences in cultural backgrounds and be associated with lower levels of education and health literacy, and a lack of familiarity with available health systems, health professionals, and support services [18, 19].

Treatment differed across sociodemographic covariables, and countries of birth, for all treatments collectively and individual treatment types. This was not explained by extent of disease at diagnosis or treatment availability. Higher socioeconomic status and diagnosis in more recent years tended to be associated with a higher likelihood of receiving treatment. Unsurprisingly, older age and comorbidities tended to be associated with not receiving treatment, regardless of extent of disease or treatment type.

In parallel with patterns of treatment, survival at one- and five-years following diagnosis was higher for people born in countries other than Australia, the United Kingdom, and Germany. Higher socioeconomic status and diagnosis in more recent years tended to be associated with a reduced risk of death (a lower HR) and older age, male sex, and comorbidities tended to be associated with an increased risk of death (a higher HR), which was not explained by the extent of disease at diagnosis. Further research is needed into the underlying causes of these differences.

During the 2003–2016 study period, NSCLC treatment options and guidelines have changed markedly [20–22]. The increasing use of multi-modality imaging and biomarkers in diagnostic workups, improved surgical techniques, advent of new systemic therapies such as PD-1/PD-L1 inhibitors, and advances in radiotherapy such as stereotactic body radiotherapy are examples [23–29]. The respective impact of these changes on survival outcomes needs further evaluation.

Study limitations included lack of access to data on systemic therapies and treatments given under a clinical trial protocol that were not yet included in the PBS. Also, we did not have access to data on public hospital treatments performed outside of NSW or treatments performed in other countries. We consider it possible that some people may have returned to their birth countries for a second opinion and treatment. A similar limitation exists with the survival results, as people may have returned to their home country prior to dying there, such that these people would be misclassified as alive in our data.

It is also possible that the comparatively higher survival seen in people born outside Australian may be partly due to the health requirements and screening of migrants at entry to Australia, which may have led to migrant groups being younger, fitter, and healthier than the Australian-born (the so-called ‘healthy migrant effect’) [30].

The use of country of birth to infer cultural differences has limitations, as it may not accurately reflect a person’s culture or ethnicity. The analyses did not account for heterogeneity between people within each country of birth group [17]. Also people from other countries may experience a change in risk over time towards that of the adopted country, but data were not available to us to investigate that aspect.

Although the need for complete and accurate data on country of birth or cultural background has been acknowledged, few data elements are currently available in routinely collected administrative datasets that would allow more in-depth analyses by ethnicity [18, 19]. Further research in this area should endeavour to use datasets that contain more comprehensive data elements on ethnicity, language proficiency, and cultural identity, as in census data held by the Australia Bureau of Statistics.

The present data indicate that variations exist in NSW in extent of disease at diagnosis, treatment, and survival from NSCLC for people born in different countries. These variations may be influenced by differences in the underlying health of migrants from different countries, cultural and social factors, differences in health behaviours (including tobacco use), and other factors. Further research is needed to investigate these and other possible explanations for variations in survival. Attempts should be made where possible to improve access to health services and better outcomes for all people diagnosed NSCLC in NSW.

## Conclusions

Variations exist in treatment and survival by country of birth in NSW. This may be affected by differences in factors not recorded in the NSW Registry, including use of health services, family histories, underlying health conditions, other intrinsic factors, and cultural, social, and behavioural influences. Means of complementing existing prognostic data with TNM and other prognostic indicators are needed, including new and emerging biomarkers. Major challenges include improving patient follow-up, as when they return to their birth countries in the terminal stages of their cancer treatment.

## Supplementary Information

**Additional file 1.** Logistic and proportional hazards regression and APDC, MBS, and PBS item code lists.

## Data Availability

The data that support the findings of this study are available from the NSW Cancer Institute and Australia Institute of Health and Welfare but restrictions apply to the availability of these data, which were used under license for the current study, and so are not publicly available. Data are however available from the authors upon reasonable request and with permission of the NSW Department of Health and Ethics Committees, contact corresponding author David Roder for additional information.
