# Supplementary material for: Country of birth and non-small cell lung cancer incidence, treatment, and outcomes in New South Wales, Australia: a population-based linkage study
Source: BMC Pulm Med. 2022 Sep 27;22:366. doi: 10.1186/s12890-022-02163-z (PMC9513895; doi:10.1186/s12890-022-02163-z)
Supplement: Supplementary file 1 — Additional file 1. Logistic and proportional hazards regression and APDC, MBS, and PBS item code lists. [file 12890_2022_2163_MOESM1_ESM.docx]

**Supplementary Table 1. APDC, MBS, and PBS item codes used for identifying NSCLC treatment**

| **Surgery – APDC procedure codes** | |
| --- | --- |
| 38438-00 | Segmental resection of lung |
| 38438-01 | Lobectomy of lung |
| 38438-02 | Pneumonectomy |
| 38440-00 | Wedge resection of lung |
| 38440-01 | Radical wedge resection of lung |
| 38441-00 | Radical lobectomy |
| 38441-01 | Radical pneumonectomy |
| 90169-00 | Endoscopic wedge resection of lung |
| **Surgery – MBS item codes** | |
| 38438 | PNEUMONECTOMY or LOBECTOMY or SEGMENTECTOMY not being a service associated with a service to which Item 38418 applies |
| 38440 | LUNG, wedge resection of |
| 38441 | RADICAL LOBECTOMY or PNEUMONECTOMY including resection of chest wall, diaphragm, pericardium, or formal mediastinal node dissection |
| **Systemic therapy – APDC diagnosis and procedure codes** | |
| Z51.1 | Pharmacotherapy session for neoplasm |
| 13915-00 | Chemotherapy, intravenous administration <= 1 hours duration |
| 13918-00 | Chemotherapy, intravenous administration > 1 hours duration and <= 6 hours duration |
| 13921-00 | Chemotherapy, intravenous administration > 6 hours duration |
| 13927-00 | Chemotherapy, intra-arterial administration |
| 13939-00 | Loading of implantable infusion device or pump |
| 13942-00 | Loading of ambulatory drug delivery device |
| 90760-00 | Chemotherapy, oral administration |
| 90767-00 | Chemotherapy, subcutaneous or intramuscular administration |
| 90768-00 | Chemotherapy, other administration |
| 96196-00 | Intra-arterial administration of pharmacological agent, antineoplastic agent |
| 96197-00 | Intramuscular administration of pharmacological agent, antineoplastic agent |
| 96198-00 | Intrathecal administration of pharmacological agent, antineoplastic agent |
| 96199-00 | Intravenous administration of pharmacological agent, antineoplastic agent |
| 96200-00 | Subcutaneous administration of pharmacological agent, antineoplastic agent |
| 96201-00 | Intracavitary administration of pharmacological agent, antineoplastic agent |
| 96202-00 | Enteral administration of pharmacological agent, antineoplastic agent |
| 96203-00 | Oral administration of pharmacological agent, antineoplastic agent |
| 96204-00 | Administration of pharmacological agent via external vascular catheter, antineoplastic agent |
| 96205-00 | Other administration of pharmacological agent, antineoplastic agent |
| 96206-00 | Unspecified administration of pharmacological agent, antineoplastic agent |
| 96207-00 | Loading of implantable infusion device or pump, antineoplastic agent (deleted from 6th edition) |
| 96208-00 | Loading of ambulatory drug delivery device, antineoplastic agent (deleted from 6th edition) |
| 96209-00 | Loading of drug delivery device, antineoplastic agent |
| **Systemic therapy – MBS item codes** | |
| 13915 | CYTOTOXIC CHEMOTHERAPY, administration of, either by intravenous push technique (directly into a vein, or a butterfly needle, or the side-arm of an infusion) or by intravenous infusion of not more than 1 hours duration - payable once only on the same day, not being a service associated with photodynamic therapy with verteporfin or for the administration of drugs used immediately prior to, or with microwave (UHF radiowave) cancer therapy alone |
| 13918 | CYTOTOXIC CHEMOTHERAPY, administration of, by intravenous infusion of more than 1 hours duration but not more than 6 hours duration - payable once only on the same day |
| 13921 | CYTOTOXIC CHEMOTHERAPY, administration of, by intravenous infusion of more than 6 hours duration - for the first day of treatment |
| 13924 | CYTOTOXIC CHEMOTHERAPY, administration of, by intravenous infusion of more than 6 hours duration - on each day subsequent to the first in the same continuous treatment episode |
| 13927 | CYTOTOXIC CHEMOTHERAPY, administration of, either by intra-arterial push technique (directly into an artery, a butterfly needle or the side-arm of an infusion) or by intra-arterial infusion of not more than 1 hours duration - payable once only on the same day |
| 13930 | CYTOTOXIC CHEMOTHERAPY, administration of, by intra-arterial infusion of more than 1 hours duration but not more than 6 hours duration - payable once only on the same day |
| 13933 | CYTOTOXIC CHEMOTHERAPY, administration of, by intra-arterial infusion of more than 6 hours duration - for the first day of treatment |
| 13936 | CYTOTOXIC CHEMOTHERAPY, administration of, by intra-arterial infusion of more than 6 hours duration - on each day subsequent to the first in the same continuous treatment episode |
| 13939 | IMPLANTED PUMP OR RESERVOIR, loading of, with a cytotoxic agent or agents, not being a service associated with a service to which item 13915, 13918, 13921, 13924, 13927, 13930, 13933, 13936 or 13945 applies |
| 13942 | AMBULATORY DRUG DELIVERY DEVICE, loading of, with a cytotoxic agent or agents for the infusion of the agent or agents via the intravenous, intra-arterial or spinal routes, not being a service associated with a service to which item 13915, 13918, 13921, 13924, 13927, 13930, 13933, 13936 or 13945 applies |
| 13945 | LONG-TERM IMPLANTED DRUG DELIVERY DEVICE FOR CYTOTOXIC CHEMOTHERAPY, accessing of |
| 13948 | CYTOTOXIC AGENT, instillation of, into a body cavity |
| **Systemic therapy – PBS ATC codes** | |
| L01 | Antineoplastic agent |
| L03 | Immunostimulant |
| L04 | Immunosuppressant |
| **Radiotherapy – APDC diagnosis and procedure codes** | |
| Z51.0 | Radiotherapy session |
| 15000-00 | Radiation treatment, superficial, 1 field |
| 15003-00 | Radiation treatment, superficial, >= 2 fields |
| 15012-00 | Brachytherapy, eye, using strontium plate |
| 15012-01 | Brachytherapy, eye, using scleral plaque |
| 15100-00 | Radiation treatment, orthovoltage, 1 field |
| 15103-00 | Radiation treatment, orthovoltage, >= 2 fields |
| 15203-00 | Radiation treatment, megavoltage, 1 field, single modality linear accelerator |
| 15204-00 | Radiation treatment, megavoltage, >=2 field, single modality linear accelerator |
| 15207-00 | Radiation treatment, megavoltage, 1 field, dual modality linear accelerator |
| 15208-00 | Radiation treatment, megavoltage, >=2 fields, dual modality linear accelerator |
| 15224-00 | Radiation treatment, megavoltage, 1 field, single modality linear accelerator |
| 15239-00 | Radiation treatment, megavoltage, >=2 field, single modality linear accelerator |
| 15254-00 | Radiation treatment, megavoltage, 1 field, dual modality linear accelerator |
| 15269-00 | Radiation treatment, megavoltage, >=2 fields, dual modality linear accelerator |
| 15303-00 | Brachytherapy, intrauterine, low dose rate |
| 15304-00 | Brachytherapy, intrauterine, high dose rate |
| 15311-00 | Brachytherapy, intravaginal, low dose rate |
| 15312-00 | Brachytherapy, intravaginal, high dose rate |
| 15319-00 | Brachytherapy, combined intrauterine and intravaginal, low dose rate |
| 15320-00 | Brachytherapy, combined intrauterine and intravaginal, high dose rate |
| 15327-00 | Brachytherapy with implantation of removable single plane, low dose rate |
| 15327-01 | Brachytherapy with implantation of removable single plane, pulsed dose rate |
| 15327-02 | Brachytherapy with implantation of removable multiple planes or volume implant, low dose rate |
| 15327-03 | Brachytherapy with implantation of removable multiple planes or volume implant, pulsed dose rate |
| 15327-04 | Brachytherapy with implantation of permanent implant, < 10 sources |
| 15327-05 | Brachytherapy with implantation of permanent implant, >= 10 sources |
| 15327-06 | Brachytherapy with implantation of removable single plane,high dose rate |
| 15327-07 | Brachytherapy with implantation of removable multiple plane, or volume implant, high dose rate |
| 15339-00 | Removal of sealed radioactive source |
| 15360-00 | Brachytherapy, intravascular |
| 15550-00 | Radiation field setting for three-dimensional conformal radiation therapy [3DCRT] |
| 15556-00 | Dosimetry by CT interfacing computer for three-dimensional conformal radiation therapy [3DCRT] |
| 15556-01 | Dosimetry by non-CT interfacing computer for three-dimensional conformal radiation therapy [3DCRT] |
| 15600-00 | Stereotactic radiation treatment, single dose |
| 15600-01 | Stereotactic radiation treatment, fractionated |
| 15600-02 | Hemi body irradiation |
| 15600-03 | Total body irradiation |
| 15600-04 | Total skin irradiation |
| 16000-00 | Administration of a therapeutic dose of other unsealed radioisotope |
| 16003-00 | Administration of a therapeutic dose of Yttrium 90 |
| 16009-00 | Administration of a therapeutic dose of Iodine 131 |
| 16012-00 | Administration of a therapeutic dose of Phosphorous 32 |
| 16015-00 | Administration of a therapeutic dose of Strontium 89 |
| 16018-00 | Administration of a therapeutic dose of 153 SM-Lexidronan |
| 90764-00 | Brachytherapy, intracavitary, low dose rate |
| 90764-01 | Brachytherapy, intracavitary, high dose rate |
| 90960-00 | Administration of a therapeutic dose of other unsealed radioisotope |
| **Radiotherapy – MBS item codes** | |
| 15100 | RADIOTHERAPY, DEEP OR ORTHOVOLTAGE each attendance at which fractionated treatment is given at 3 or more treatments per week - 1 field |
| 15103 | - 2 or more fields up to a maximum of 5 additional fields (rotational therapy being 3 fields) |
| 15106 | RADIOTHERAPY, DEEP OR ORTHOVOLTAGEeach attendance at which fractionated treatment is given at 2 treatments per week or less frequently - 1 field |
| 15109 | - 2 or more fields up to a maximum of 5 additional fields (rotational therapy being 3 fields) |
| 15112 | RADIOTHERAPY, DEEP OR ORTHOVOLTAGEattendance at which single dose technique is applied 1 field |
| 15115 | - 2 or more fields up to a maximum of 5 additional fields (rotational therapy being 3 fields) |
| 15211 | RADIATION ONCOLOGY TREATMENT, using cobalt unit or caesium teletherapy uniteach attendance at which treatment is given - 1 field |
| 15214 | - 2 or more fields up to a maximum of 5 additional fields (rotational therapy being 3 fields) |
| 15215 | RADIATION ONCOLOGY TREATMENT, using a single photon energy linear accelerator with or without electron facilities - each attendance at which treatment is given - 1 field - treatment delivered to primary site (lung) |
| 15224 | RADIATION ONCOLOGY TREATMENT, using a single photon energy linear accelerator with or without electron facilities - each attendance at which treatment is given - 1 field - treatment delivered to primary site for diseases and conditions not covered by items 15215, 15218 and 15221 |
| 15227 | RADIATION ONCOLOGY TREATMENT, using a single photon energy linear accelerator with or without electron facilities - each attendance at which treatment is given - 1 field - treatment delivered to secondary site |
| 15230 | RADIATION ONCOLOGY TREATMENT, using a single photon energy linear accelerator with or without electron facilities - each attendance at which treatment is given - 2 or more fields up to a maximum of 5 additional fields (rotational therapy being 3 fields) - treatment delivered to primary site (lung) |
| 15239 | RADIATION ONCOLOGY TREATMENT, using a single photon energy linear accelerator with or without electron facilities - each attendance at which treatment is given - 2 or more fields up to a maximum of 5 additional fields (rotational therapy being 3 fields) - treatment delivered to primary site for diseases and conditions not covered by items 15230, 15233 or 15236 |
| 15242 | RADIATION ONCOLOGY TREATMENT, using a single photon energy linear accelerator with or without electron facilities - each attendance at which treatment is given - 2 or more fields up to a maximum of 5 additional fields (rotational therapy being 3 fields) - treatment delivered to secondary site |
| 15245 | RADIATION ONCOLOGY TREATMENT, using a dual photon energy linear accelerator with a minimum higher energy of at least 10MV photons, with electron facilities - each attendance at which treatment is given - 1 field - treatment delivered to primary site (lung) |
| 15254 | RADIATION ONCOLOGY TREATMENT, using a dual photon energy linear accelerator with a minimum higher energy of at least 10MV photons, with electron facilities - each attendance at which treatment is given - 1 field - treatment delivered to primary site for diseases and conditions not covered by items 15245, 15248 or 15251 |
| 15257 | RADIATION ONCOLOGY TREATMENT, using a dual photon energy linear accelerator with a minimum higher energy of at least 10MV photons, with electron facilities - each attendance at which treatment is given - 1 field - treatment delivered to secondary site |
| 15260 | RADIATION ONCOLOGY TREATMENT, using a dual photon energy linear accelerator with a minimum higher energy of at least 10MV photons, with electron facilities - each attendance at which treatment is given - 2 or more fields up to a maximum of 5 additional fields (rotational therapy being 3 fields) - treatment delivered to primary site (lung) |
| 15269 | RADIATION ONCOLOGY TREATMENT, using a dual photon energy linear accelerator with a minimum higher energy of at least 10MV photons, with electron facilities - each attendance at which treatment is given - 2 or more fields up to a maximum of 5 additional fields (rotational therapy being 3 fields) - treatment delivered to primary site for diseases and conditions not covered by items 15260, 15263 or 15266 |
| 15272 | RADIATION ONCOLOGY TREATMENT, using a dual photon energy linear accelerator with a minimum higher energy of at least 10MV photons, with electron facilities - each attendance at which treatment is given - 2 or more fields up to a maximum of 5 additional fields (rotational therapy being 3 fields) - treatment delivered to secondary site |
| 15275 | RADIATION ONCOLOGY TREATMENT with IGRT imaging facilities undertaken: (a) to implement an IMRT dosimetry plan prepared in accordance with item 15565; and (b) utilising an intensity modulated treatment delivery mode (delivered by a fixed or dynamic gantry linear accelerator or by a helical non C-arm based linear accelerator), once only at each attendance at which treatment is given. |
| 15303 | INTRAUTERINE TREATMENT ALONE using radioactive sealed sources having a half-life greater than 115 days using manual afterloading techniques |
| 15304 | INTRAUTERINE TREATMENT ALONE using radioactive sealed sources having a half-life greater than 115 days using automatic afterloading techniques |
| 15307 | INTRAUTERINE TREATMENT ALONE using radioactive sealed sources having a half-life of less than 115 days including iodine, gold, iridium or tantalum using manual afterloading techniques |
| 15308 | INTRAUTERINE TREATMENT ALONE using radioactive sealed sources having a half-life of less than 115 days including iodine, gold, iridium or tantalum using automatic afterloading techniques |
| 15311 | INTRAVAGINAL TREATMENT ALONE using radioactive sealed sources having a half-life greater than 115 days using manual afterloading techniques |
| 15312 | INTRAVAGINAL TREATMENT ALONE using radioactive sealed sources having a half-life greater than 115 days using automatic afterloading techniques |
| 15315 | INTRAVAGINAL TREATMENT ALONE using radioactive sealed sources having a half-life of less than 115 days including iodine, gold, iridium or tantalum using manual afterloading techniques |
| 15316 | INTRAVAGINAL TREATMENT ALONE using radioactive sealed sources having a half-life of less than 115 days including iodine, gold, iridium or tantalum using automatic afterloading techniques |
| 15319 | COMBINED INTRAUTERINE AND INTRAVAGINAL TREATMENT using radioactive sealed sources having a half-life greater than 115 days using manual afterloading techniques |
| 15320 | COMBINED INTRAUTERINE AND INTRAVAGINAL TREATMENT using radioactive sealed sources having a half-life greater than 115 days using automatic afterloading techniques |
| 15323 | COMBINED INTRAUTERINE AND INTRAVAGINAL TREATMENT using radioactive sealed sources having a half-life of less than 115 days including iodine, gold, iridium or tantalum using manual afterloading techniques |
| 15324 | COMBINED INTRAUTERINE AND INTRAVAGINAL TREATMENT using radioactive sealed sources having a half-life of less than 115 days including iodine, gold, iridium or tantalum using automatic afterloading techniques |
| 15327 | IMPLANTATION OF A SEALED RADIOACTIVE SOURCE (having a half-life of less than 115 days including iodine, gold, iridium or tantalum) to a region, under general anaesthesia, or epidural or spinal (intrathecal) nerve block, requiring surgical exposure and using manual afterloading techniques |
| 15328 | IMPLANTATION OF A SEALED RADIOACTIVE SOURCE (having a half-life of less than 115 days including iodine, gold, iridium or tantalum) to a region, under general anaesthesia, or epidural or spinal (intrathecal) nerve block, requiring surgical exposure and using automatic afterloading techniques |
| 15331 | IMPLANTATION OF A SEALED RADIOACTIVE SOURCE (having a half-life of less than 115 days including iodine, gold, iridium or tantalum) to a site (including the tongue, mouth, salivary gland, axilla, subcutaneous sites), where the volume treated involves multiple planes but does not require surgical exposure and using manual afterloading techniques |
| 15332 | IMPLANTATION OF A SEALED RADIOACTIVE SOURCE (having a half-life of less than 115 days including iodine, gold, iridium or tantalum) to a site (including the tongue, mouth, salivary gland, axilla, subcutaneous sites), where the volume treated involves multiple planes but does not require surgical exposure and using automatic afterloading techniques |
| 15335 | IMPLANTATION OF A SEALED RADIOACTIVE SOURCE (having a half-life of less than 115 days including iodine, gold, iridium or tantalum) to a site where the volume treated involves only a single plane but does not require surgical exposure and using manual afterloading techniques |
| 15336 | IMPLANTATION OF A SEALED RADIOACTIVE SOURCE (having a half-life of less than 115 days including iodine, gold, iridium or tantalum) to a site where the volume treated involves only a single plane but does not require surgical exposure and using automatic afterloading techniques |
| 15342 | CONSTRUCTION AND APPLICATION OF A RADIOACTIVE MOULD using a sealed source having a half-life of greater than 115 days, to treat intracavity, intraoral or intranasal site |
| 15345 | CONSTRUCTION AND APPLICATION OF A RADIOACTIVE MOULD using a sealed source having a half-life of less than 115 days including iodine, gold, iridium or tantalum to treat intracavity, intraoral or intranasal sites |
| 15348 | SUBSEQUENT APPLICATIONS OF RADIOACTIVE MOULD referred to in item 15342 or 15345each attendance |
| 15351 | CONSTRUCTION WITH OR WITHOUT INITIAL APPLICATION OF RADIOACTIVE MOULD not exceeding 5 cm. diameter to an external surface |
| 15354 | CONSTRUCTION AND INITIAL APPLICATION OF RADIOACTIVE MOULD 5 cm. or more in diameter to an external surface |
| 15357 | SUBSEQUENT APPLICATIONS OF RADIOACTIVE MOULD referred to in item 15351 or 15354each attendance |
| 15360 | CATHETER BASED INTRAVASCULAR BRACHYTHERAPY for the treatment of in-stent restenoses of 1 coronary artery, administration of radioactive sealed sources having a half life of less than 115 days using automated intravascular brachytherapy systems approved by the Therapeutic Goods Administration. The procedure must be performed by a radiation oncologist in association with a cardiologist and be associated with a service to which item 38321, 38324, 38327 or 38330 applies. |
| 15363 | CATHETER BASED INTRAVASCULAR BRACHYTHERAPY for the treatment of in-stent restenoses of 1 coronary artery, administration of radioactive sealed sources having a half life of greater than 115 days using automated intravascular brachytherapy systems approved by the Therapeutic Goods Administration. The procedure must be performed by a radiation oncologist in association with a cardiologist and be associated with a service to which item 38321, 38324, 38327 or 38330 applies. |
| 15600 | STEREOTACTIC RADIOSURGERY, including all radiation oncology consultations, planning, simulation, dosimetry and treatment |

**Supplementary Table 2. Logistic regression output for receipt of any treatment within 12 months of diagnosis, NSCLC, 2003-2016**

|  | **Odds ratio (95% CI)** | | | |
| --- | --- | --- | --- | --- |
| **Characteristic** | **All NSCLC** | **Localised** | **Regional** | **Distant** |
| *Country of birth* | | | | |
| Australia (reference) | 1.00 | 1.00 | 1.00 | 1.00 |
| New Zealand | 1.02 (0.79-1.32) | 0.95 (0.51-1.87) | 1.59 (0.83-3.04) | 0.95 (0.69-1.31) |
| United Kingdom | **1.22 (1.10-1.36)** | **1.30 (1.03-1.68)** | 1.19 (0.93-1.54) | **1.25 (1.09-1.44)** |
| Other English-speaking | **1.42 (1.07-1.91)** | 1.27 (0.73-2.40) | **4.25 (1.66-10.90)** | 1.17 (0.82-1.71) |
| China | 1.15 (0.96-1.39) | 0.92 (0.59-1.44) | **1.63 (1.02-2.61)** | 1.19 (0.95-1.51) |
| Philippines | 1.38 (0.93-2.13) | 1.28 (0.53-3.80) | 0.92 (0.37-2.28) | **1.66 (1.03-2.93)** |
| Vietnam | 1.09 (0.81-1.50) | 1.31 (0.66-3.04) | **0.49 (0.25-0.96)** | 1.42 (0.98-2.15) |
| Germany | 1.01 (0.78-1.33) | 1.02 (0.54-2.02) | 0.82 (0.46-1.46) | 1.15 (0.82-1.64) |
| Greece | **1.61 (1.29-2.04)** | 1.46 (0.88-2.64) | 1.64 (1.00-2.70) | **1.61 (1.21-2.21)** |
| Italy | **1.51 (1.27-1.82)** | **1.50 (1.04-2.25)** | **1.88 (1.21-2.92)** | **1.39 (1.10-1.79)** |
| Lebanon | **1.54 (1.18-2.07)** | 1.17 (0.67-2.23) | 1.71 (0.86-3.42) | **1.63 (1.15-2.41)** |
| Other non-English speaking | **1.15 (1.05-1.26)** | 1.11 (0.90-1.37) | 1.14 (0.91-1.41) | **1.19 (1.06-1.35)** |
| *Age at diagnosis* | | | | |
| Continuous (1+ year) | **0.92 (0.92-0.92)** | **0.91 (0.90-0.92)** | **0.91 (0.90-0.91)** | **0.92 (0.92-0.93)** |
| *Sex* | | | | |
| Female | 1.00 | 1.00 | 1.00 | 1.00 |
| Male | 0.97 (0.91-1.03) | 0.91 (0.79-1.05) | 1.02 (0.88-1.19) | 0.98 (0.90-1.07) |
| *Year of diagnosis* | | | | |
| Continuous (2003-2016) | **1.04 (1.04-1.05)** | **1.04 (1.02-1.05)** | **1.05 (1.03-1.07)** | **1.05 (1.04-1.06)** |
| *Charlson score* | | | | |
| 0 | 1.00 | 1.00 | 1.00 | 1.00 |
| >= 1 | **0.39 (0.37-0.42)** | **0.36 (0.31-0.42)** | **0.35 (0.30-0.41)** | **0.40 (0.36-0.44)** |
| *SEIFA* | | | | |
| Quintile 1 | 1.00 | 1.00 | 1.00 | 1.00 |
| Quintile 2 | **1.20 (1.10-1.31)** | 1.12 (0.93-1.35) | **1.26 (1.03-1.54)** | **1.18 (1.06-1.32)** |
| Quintile 3 | **1.27 (1.17-1.40)** | **1.31 (1.08-1.62)** | **1.38 (1.12-1.71)** | **1.20 (1.07-1.35)** |
| Quintile 4 | **1.53 (1.39-1.69)** | **1.55 (1.25-1.96)** | **1.52 (1.20-1.92)** | **1.51 (1.33-1.72)** |
| Quintile 5 | **1.91 (1.72-2.13)** | **1.72 (1.37-2.21)** | **1.70 (1.31-2.19)** | **2.08 (1.81-2.40)** |

**Notes:**
1. Each characteristic has a reference value at OR = 1.00.

2. SEIFA Quintile 1 = most disadvantaged.

3. Potential non-random differences are bolded.

**Supplementary Table 3. Logistic regression output for receipt of surgery within 12 months of diagnosis, NSCLC, 2003-2016**

|  | **Odds ratio (95% CI)** | | |
| --- | --- | --- | --- |
| **Characteristic** | **All NSCLC** | **Localised** | **Regional** |
| *Country of birth* | | | |
| Australia (reference) | 1.00 | 1.00 | 1.00 |
| New Zealand | 0.92 (0.72-1.18) | 1.07 (0.63-1.85) | 0.97 (0.64-1.46) |
| United Kingdom | 1.07 (0.95-1.20) | 1.22 (0.99-1.52) | 1.08 (0.89-1.33) |
| Other English-speaking | **1.77 (1.39-2.29)** | 1.58 (0.98-2.70) | **1.97 (1.26-3.28)** |
| China | 1.11 (0.92-1.33) | 1.01 (0.69-1.49) | **1.71 (1.26-2.38)** |
| Philippines | 1.04 (0.73-1.46) | 1.43 (0.70-3.27) | 1.54 (0.82-3.14) |
| Vietnam | **0.71 (0.49-0.98)** | 0.83 (0.42-1.53) | 1.04 (0.55-1.90) |
| Germany | 0.80 (0.57-1.10) | 0.92 (0.48-1.67) | 1.05 (0.63-1.72) |
| Greece | **1.37 (1.09-1.73)** | 1.45 (0.92-2.39) | **1.66 (1.17-2.42)** |
| Italy | **1.56 (1.29-1.88)** | 1.35 (0.98-1.90) | **1.96 (1.43-2.76)** |
| Lebanon | 1.14 (0.86-1.49) | 1.48 (0.93-2.49) | 0.77 (0.44-1.26) |
| Other non-English speaking | **1.13 (1.03-1.25)** | 1.17 (0.98-1.41) | **1.30 (1.11-1.55)** |
| *Age at diagnosis* | | | |
| Continuous (1+ year) | **0.98 (0.97-0.98)** | **0.94 (0.93-0.95)** | **0.97 (0.96-0.97)** |
| *Sex* | | | |
| Female | 1.00 | 1.00 | 1.00 |
| Male | **0.81 (0.76-0.86)** | **0.76 (0.67-0.86)** | **0.80 (0.71-0.90)** |
| *Year of diagnosis* | | | |
| Continuous (2003-2016) | **1.01 (1.01-1.02)** | **1.05 (1.04-1.07)** | 1.01 (0.99-1.02) |
| *Charlson score* | | | |
| 0 | 1.00 | 1.00 | 1.00 |
| >= 1 | **0.62 (0.56-0.68)** | **0.43 (0.37-0.50)** | **0.57 (0.48-0.67)** |
| *SEIFA* | | | |
| Quintile 1 | 1.00 | 1.00 | 1.00 |
| Quintile 2 | **1.21 (1.10-1.33)** | 1.12 (0.95-1.33) | **1.24 (1.05-1.46)** |
| Quintile 3 | **1.26 (1.14-1.39)** | **1.33 (1.12-1.61)** | **1.23 (1.04-1.47)** |
| Quintile 4 | **1.25 (1.13-1.39)** | **1.32 (1.09-1.61)** | **1.33 (1.11-1.59)** |
| Quintile 5 | **1.52 (1.37-1.69)** | **1.72 (1.41-2.12)** | **1.53 (1.27-1.85)** |

**Notes:**
1. Each characteristic has a reference value at OR = 1.00.

2. SEIFA Quintile 1 = most disadvantaged.

3. Potential non-random differences are bolded.

**Supplementary Table 4. Logistic regression output for receipt of systemic therapy within 12 months of diagnosis, NSCLC, 2003-2016**

|  | **Odds ratio (95% CI)** | | | |
| --- | --- | --- | --- | --- |
| **Characteristic** | **All NSCLC** | **Localised** | **Regional** | **Distant** |
| *Country of birth* | | | | |
| Australia (reference) | 1.00 | 1.00 | 1.00 | 1.00 |
| New Zealand | 1.03 (0.83-1.28) | 1.37 (0.79-2.39) | 1.05 (0.67-1.65) | 0.86 (0.65-1.15) |
| United Kingdom | **1.14 (1.03-1.26)** | 1.04 (0.80-1.35) | 1.09 (0.89-1.35) | **1.20 (1.04-1.38)** |
| Other English-speaking | 1.13 (0.88-1.45) | 0.66 (0.35-1.25) | 1.26 (0.75-2.18) | 1.34 (0.96-1.92) |
| China | **1.25 (1.06-1.48)** | **0.58 (0.35-0.96)** | 1.28 (0.90-1.83) | **1.52 (1.22-1.92)** |
| Philippines | **1.51 (1.10-2.13)** | 0.44 (0.17-1.14) | 1.66 (0.81-4.09) | **2.18 (1.44-3.57)** |
| Vietnam | 1.17 (0.89-1.54) | 1.23 (0.62-2.44) | 0.63 (0.30-1.19) | 1.39 (0.99-1.98) |
| Germany | 1.10 (0.85-1.43) | 0.78 (0.36-1.68) | 1.03 (0.62-1.72) | 1.15 (0.81-1.63) |
| Greece | **1.79 (1.47-2.21)** | 1.40 (0.83-2.38) | **1.48 (1.02-2.20)** | **2.02 (1.53-2.72)** |
| Italy | **1.27 (1.07-1.52)** | 1.03 (0.69-1.54) | 1.10 (0.77-1.58) | **1.61 (1.26-2.08)** |
| Lebanon | 1.18 (0.93-1.50) | **0.41 (0.20-0.85)** | 1.46 (0.90-2.48) | **1.55 (1.12-2.20)** |
| Other non-English speaking | 1.06 (0.97-1.16) | 0.88 (0.70-1.10) | 1.04 (0.87-1.25) | **1.15 (1.03-1.30)** |
| *Age at diagnosis* | | | | |
| Continuous (1+ year) | **0.92 (0.92-0.93)** | **0.95 (0.94-0.95)** | **0.91 (0.90-0.91)** | **0.92 (0.92-0.93)** |
| *Sex* | | | | |
| Female | 1.00 | 1.00 | 1.00 | 1.00 |
| Male | 1.03 (0.97-1.09) | **1.27 (1.09-1.49)** | 1.02 (0.90-1.16) | 0.92 (0.84-1.00) |
| *Year of diagnosis* | | | | |
| Continuous (2003-2016) | **1.03 (1.02-1.04)** | **0.96 (0.95-0.98)** | **1.05 (1.04-1.07)** | **1.04 (1.03-1.05)** |
| *Charlson score* | | | | |
| 0 | 1.00 | 1.00 | 1.00 | 1.00 |
| >= 1 | **0.42 (0.39-0.46)** | **0.67 (0.56-0.81)** | **0.43 (0.36-0.50)** | **0.38 (0.34-0.42)** |
| *SEIFA* | | | | |
| Quintile 1 | 1.00 | 1.00 | 1.00 | 1.00 |
| Quintile 2 | **1.10 (1.01-1.19)** | 0.92 (0.75-1.15) | **1.26 (1.07-1.50)** | 1.11 (0.99-1.24) |
| Quintile 3 | **1.26 (1.15-1.37)** | 1.15 (0.92-1.44) | 1.18 (1.00-1.42) | **1.33 (1.19-1.50)** |
| Quintile 4 | **1.59 (1.45-1.74)** | **1.47 (1.17-1.85)** | **1.46 (1.21-1.77)** | **1.74 (1.54-1.98)** |
| Quintile 5 | **2.02 (1.84-2.23)** | **1.43 (1.13-1.82)** | **1.68 (1.38-2.07)** | **2.63 (2.31-3.01)** |

**Notes:**
1. Each characteristic has a reference value at OR = 1.00.

2. SEIFA Quintile 1 = most disadvantaged.

3. Potential non-random differences are bolded.

**Supplementary Table 5. Logistic regression output for receipt of radiotherapy within 12 months of diagnosis, NSCLC, 2003-2016**

|  | **Odds ratio (95% CI)** | | | |
| --- | --- | --- | --- | --- |
| **Characteristic** | **All NSCLC** | **Localised** | **Regional** | **Distant** |
| *Country of birth* | | | | |
| Australia (reference) | 1.00 | 1.00 | 1.00 | 1.00 |
| New Zealand | 1.08 (0.88-1.32) | 0.77 (0.38-1.37) | 1.09 (0.74-1.62) | 1.06 (0.81-1.40) |
| United Kingdom | **1.15 (1.05-1.26)** | 1.11 (0.88-1.39) | 1.13 (0.93-1.36) | **1.17 (1.03-1.33)** |
| Other English-speaking | 0.79 (0.62-1.00) | 0.65 (0.32-1.14) | 0.80 (0.49-1.27) | 0.86 (0.62-1.19) |
| China | **0.73 (0.62-0.85)** | **0.64 (0.38-0.98)** | 0.75 (0.54-1.02) | **0.72 (0.58-0.89)** |
| Philippines | 0.86 (0.63-1.16) | 0.70 (0.25-1.50) | 0.81 (0.40-1.54) | 0.89 (0.60-1.32) |
| Vietnam | 1.04 (0.81-1.33) | 1.32 (0.69-2.50) | 0.72 (0.39-1.25) | 1.02 (0.74-1.41) |
| Germany | 1.06 (0.83-1.35) | 1.12 (0.57-2.08) | 0.82 (0.50-1.30) | 1.12 (0.81-1.54) |
| Greece | 1.20 (0.99-1.46) | 0.67 (0.35-1.13) | 1.35 (0.96-1.95) | 1.24 (0.95-1.63) |
| Italy | 1.07 (0.91-1.26) | 0.94 (0.64-1.35) | 1.09 (0.79-1.50) | 1.20 (0.96-1.52) |
| Lebanon | 1.21 (0.97-1.51) | 0.83 (0.45-1.40) | 1.41 (0.91-2.28) | **1.37 (1.01-1.88)** |
| Other non-English speaking | 0.98 (0.91-1.06) | 0.89 (0.72-1.08) | 0.87 (0.74-1.01) | 1.07 (0.96-1.20) |
| *Age at diagnosis* | | | | |
| Continuous (1+ year) | **0.97 (0.96-0.97)** | 1.00 (0.99-1.00) | **0.97 (0.97-0.98)** | **0.96 (0.95-0.96)** |
| *Sex* | | | | |
| Female | 1.00 | 1.00 | 1.00 | 1.00 |
| Male | **1.13 (1.07-1.19)** | **1.30 (1.13-1.50)** | **1.19 (1.06-1.33)** | 1.01 (0.93-1.09) |
| *Year of diagnosis* | | | | |
| Continuous (2003-2016) | **1.03 (1.02-1.03)** | 1.00 (0.98-1.01) | **1.02 (1.01-1.04)** | **1.04 (1.03-1.05)** |
| *Charlson score* | | | | |
| 0 | 1.00 | 1.00 | 1.00 | 1.00 |
| >= 1 | **0.60 (0.56-0.65)** | 0.91 (0.77-1.06) | **0.64 (0.55-0.74)** | **0.54 (0.49-0.59)** |
| *SEIFA* | | | | |
| Quintile 1 | 1.00 | 1.00 | 1.00 | 1.00 |
| Quintile 2 | 1.03 (0.95-1.11) | 0.93 (0.77-1.11) | 0.98 (0.84-1.14) | 1.11 (1.00-1.23) |
| Quintile 3 | 0.98 (0.91-1.06) | 0.91 (0.74-1.11) | 0.95 (0.81-1.11) | 1.03 (0.92-1.15) |
| Quintile 4 | **1.14 (1.05-1.25)** | 1.05 (0.85-1.29) | 1.13 (0.96-1.34) | **1.18 (1.05-1.32)** |
| Quintile 5 | 1.08 (0.99-1.18) | 0.90 (0.72-1.13) | 0.94 (0.79-1.12) | **1.24 (1.10-1.41)** |

**Notes:**
1. Each characteristic has a reference value at OR = 1.00.

2. SEIFA Quintile 1 = most disadvantaged.

3. Potential non-random differences are bolded.

**Supplementary Table 6. Proportional hazards regression output for survival at one year following diagnosis, NSCLC, 2003-2016**

|  | **Hazard ratio (95% CI)** | | | |
| --- | --- | --- | --- | --- |
| **Characteristic** | **All NSCLC** | **Localised** | **Regional** | **Distant** |
| *Country of birth* | | | | |
| Australia (reference) | 1.00 | 1.00 | 1.00 | 1.00 |
| New Zealand | 1.01 (0.89-1.16) | 0.96 (0.59-1.58) | 0.81 (0.58-1.14) | 1.04 (0.90-1.21) |
| United Kingdom | 0.98 (0.92-1.03) | 0.89 (0.75-1.05) | 0.88 (0.76-1.01) | 0.99 (0.92-1.05) |
| Other English-speaking | **0.81 (0.70-0.95)** | 0.83 (0.55-1.27) | **0.52 (0.33-0.82)** | 0.93 (0.78-1.11) |
| China | **0.68 (0.61-0.75)** | **0.49 (0.33-0.74)** | **0.51 (0.37-0.69)** | **0.65 (0.57-0.73)** |
| Philippines | **0.69 (0.55-0.86)** | 0.54 (0.24-1.20) | 0.89 (0.52-1.54) | **0.57 (0.44-0.73)** |
| Vietnam | **0.72 (0.61-0.86)** | **0.41 (0.19-0.86)** | 0.63 (0.38-1.05) | **0.64 (0.53-0.78)** |
| Germany | 1.06 (0.92-1.23) | 0.97 (0.61-1.55) | 1.02 (0.72-1.44) | 0.99 (0.84-1.18) |
| Greece | **0.71 (0.63-0.81)** | 0.66 (0.44-1.00) | **0.56 (0.42-0.76)** | **0.74 (0.64-0.85)** |
| Italy | **0.74 (0.66-0.82)** | **0.69 (0.52-0.91)** | **0.66 (0.51-0.85)** | **0.80 (0.70-0.90)** |
| Lebanon | **0.84 (0.73-0.97)** | 0.77 (0.50-1.21) | 0.74 (0.50-1.08) | 0.91 (0.77-1.07) |
| Other non-English speaking | **0.83 (0.79-0.87)** | **0.80 (0.69-0.94)** | **0.69 (0.60-0.78)** | **0.83 (0.78-0.88)** |
| *Age at diagnosis* | | | | |
| Continuous (1+ year) | **1.03 (1.02-1.03)** | **1.06 (1.05-1.06)** | **1.05 (1.04-1.05)** | **1.03 (1.02-1.03)** |
| *Sex* | | | | |
| Female | 1.00 | 1.00 | 1.00 | 1.00 |
| Male | **1.20 (1.16-1.25)** | **1.30 (1.18-1.44)** | **1.20 (1.10-1.31)** | **1.19 (1.14-1.24)** |
| *Year of diagnosis* | | | | |
| Continuous (2003-2016) | **0.97 (0.97-0.98)** | **0.94 (0.93-0.95)** | **0.95 (0.94-0.96)** | **0.98 (0.97-0.98)** |
| *Charlson score* | | | | |
| 0 | 1.00 | 1.00 | 1.00 | 1.00 |
| >= 1 | **1.50 (1.44-1.56)** | **1.84 (1.67-2.04)** | **1.87 (1.71-2.05)** | **1.53 (1.46-1.60)** |
| *SEIFA* | | | | |
| Quintile 1 | 1.00 | 1.00 | 1.00 | 1.00 |
| Quintile 2 | **0.91 (0.87-0.95)** | **0.85 (0.75-0.97)** | **0.84 (0.75-0.94)** | 0.99 (0.94-1.04) |
| Quintile 3 | **0.89 (0.85-0.94)** | **0.82 (0.71-0.94)** | 0.98 (0.87-1.10) | 0.90 (0.85-0.95) |
| Quintile 4 | **0.86 (0.82-0.91)** | **0.75 (0.64-0.88)** | 0.96 (0.84-1.09) | 0.84 (0.79-0.90) |
| Quintile 5 | **0.78 (0.74-0.82)** | **0.74 (0.62-0.87)** | **0.83 (0.72-0.95)** | 0.75 (0.70-0.80) |

**Notes:**
1. Each characteristic has a reference value at HR = 1.00.

2. SEIFA Quintile 1 = most disadvantaged.

3. Potential non-random differences are bolded.

**Supplementary Table 7. Proportional hazards regression output for survival at five years following diagnosis, NSCLC, 2003-2016**

|  | **Hazard ratio (95% CI)** | | | |
| --- | --- | --- | --- | --- |
| **Characteristic** | **All NSCLC** | **Localised** | **Regional** | **Distant** |
| *Country of birth* | | | | |
| Australia (reference) | 1.00 | 1.00 | 1.00 | 1.00 |
| New Zealand | 0.97 (0.87-1.09) | 0.95 (0.68-1.32) | 0.87 (0.69-1.09) | 1.00 (0.87-1.15) |
| United Kingdom | 0.99 (0.95-1.04) | 0.92 (0.81-1.04) | 0.92 (0.83-1.02) | 1.02 (0.96-1.08) |
| Other English-speaking | **0.79 (0.70-0.90)** | 0.73 (0.54-1.00) | **0.75 (0.57-0.99)** | 0.86 (0.73-1.02) |
| China | **0.78 (0.72-0.85)** | **0.70 (0.54-0.89)** | **0.66 (0.55-0.80)** | **0.70 (0.63-0.78)** |
| Philippines | **0.81 (0.68-0.96)** | **0.49 (0.27-0.88)** | 1.04 (0.72-1.52) | **0.68 (0.55-0.83)** |
| Vietnam | **0.85 (0.74-0.97)** | 0.68 (0.45-1.03) | 0.86 (0.63-1.19) | **0.70 (0.60-0.82)** |
| Germany | 1.05 (0.92-1.19) | 0.92 (0.66-1.30) | 1.02 (0.79-1.33) | 1.00 (0.86-1.17) |
| Greece | **0.76 (0.68-0.84)** | **0.66 (0.49-0.89)** | **0.66 (0.54-0.81)** | **0.79 (0.69-0.90)** |
| Italy | **0.75 (0.69-0.82)** | **0.81 (0.67-0.98)** | **0.64 (0.53-0.77)** | **0.79 (0.71-0.88)** |
| Lebanon | **0.82 (0.72-0.92)** | **0.67 (0.48-0.93)** | 0.81 (0.63-1.05) | 0.91 (0.78-1.07) |
| Other non-English speaking | **0.86 (0.82-0.89)** | **0.86 (0.77-0.95)** | **0.73 (0.66-0.80)** | **0.85 (0.80-0.89)** |
| *Age at diagnosis* | | | | |
| Continuous (1+ year) | **1.02 (1.02-1.02)** | **1.05 (1.04-1.05)** | **1.03 (1.03-1.04)** | **1.02 (1.02-1.02)** |
| *Sex* | | | | |
| Female | 1.00 | 1.00 | 1.00 | 1.00 |
| Male | **1.21 (1.18-1.25)** | **1.35 (1.25-1.46)** | **1.24 (1.16-1.32)** | **1.17 (1.13-1.21)** |
| *Year of diagnosis* | | | | |
| Continuous (2003-2016) | **0.97 (0.97-0.97)** | **0.94 (0.94-0.95)** | **0.95 (0.95-0.96)** | **0.97 (0.97-0.98)** |
| *Charlson score* | | | | |
| 0 | 1.00 | 1.00 | 1.00 | 1.00 |
| >= 1 | **1.38 (1.34-1.43)** | **1.62 (1.50-1.75)** | **1.56 (1.45-1.68)** | **1.43 (1.37-1.50)** |
| *SEIFA* | | | | |
| Quintile 1 | 1.00 | 1.00 | 1.00 | 1.00 |
| Quintile 2 | **0.92 (0.88-0.95)** | **0.89 (0.80-0.98)** | **0.89 (0.81-0.96)** | 0.98 (0.94-1.03) |
| Quintile 3 | **0.91 (0.87-0.95)** | **0.83 (0.75-0.92)** | 0.96 (0.88-1.05) | **0.93 (0.88-0.98)** |
| Quintile 4 | **0.88 (0.84-0.92)** | **0.79 (0.70-0.88)** | 0.94 (0.85-1.03) | **0.86 (0.82-0.91)** |
| Quintile 5 | **0.83 (0.79-0.87)** | **0.77 (0.68-0.87)** | **0.83 (0.75-0.92)** | **0.78 (0.74-0.83)** |

**Notes:**
1. Each characteristic has a reference value at HR = 1.00.

2. SEIFA Quintile 1 = most disadvantaged.

3. Potential non-random differences are bolded.
